# Amplification of the power of network hubs and degree skewness over infectious disease spread during lulls

**DOI:** 10.1371/journal.pone.0322687

**Published:** 2025-11-13

**Authors:** Benjamin Cornwell, Shiyu Ji, Shane G. Henderson, Gen Meredith

**Affiliations:** 1 Department of Sociology, Cornell University, Ithaca, New York, United States of America; 2 Department of Operations Research and Information Engineering, Cornell University, Ithaca, New York, United States of America; 3 Department of Public and Ecosystem Health, Cornell University, Ithaca, New York, United States of America; Georgetown University Medical Center, UNITED STATES OF AMERICA

## Abstract

Just as individuals’ personal social network connections shape their susceptibility to disease, the structure of larger networks in communities shapes the extent of disease spread. We examine how heterogeneity in network structure at the population level affects the spread of disease – namely, COVID-19 – considering varying levels of disease transmissibility and in-person contact rates. Using dynamic simulations that take into account network structure, social contact rates given contextual features of the community (informed by real-life data on family local family structure, schools, workplaces, and daily shopping activities), and disease infection rates, we first confirm that the presence of network hubs and high network degree skewness results in a higher level of infected peak prevalence with infectious diseases such as COVID-19 during periods of low to moderate transmissibility. However, this effect is amplified during lulls in disease spread and is suppressed during periods of greater transmissibility, rendering social network structure more significant during lulls. Moreover, in the case of already highly transmissible diseases, the role of hubs and severe degree skewness is already more continually suppressed.

## Introduction

The ability of an infectious disease to wind its way through vast and complex social networks determines how disruptive and deadly its sweep through the population will be. Social epidemiologists have spent decades pinpointing the structural features of the network systems that facilitate, condition, and channel the spread of infectious diseases throughout the population. This work has led to valuable insights into the role various aspects of social structure play in the genesis of epidemics, demystifying their ability to move ineffective through communities, and inching us closer to the prospects of early detection, manipulation, and hopefully mitigation.

There are numerous studies on this empirical connection [1–4], including on such specific issues as the link between levels of social connectedness (conceptualized in various ways) and the common cold, functional health, cognitive health, and mortality. Much of this work focuses on individual-level behaviors and the functions and effects of their social ties, including social support, coping, access to resources and social influence [5–14]. Related work elevates the focus to community-level risks and outcomes, such as the epidemic spread of disease and attendant public health policies [2,15]. Along these lines, this paper focuses on a particular health threat, which is the presence of rapidly spreading viruses that lead to the epidemic spread of deadly diseases, such as the human immunodeficiency virus (HIV), Influenza, the respiratory syncytial virus (RSV), and severe acute respiratory syndrome coronavirus 2 (SARS-CoV-2). Given the recent spate of such disease epidemics around the world (some of them ongoing or recurring), scientists have also sought to understand the factors affecting their genesis, partly in an effort to inform prevention strategies, and in an urgent effort to reduce morbidity and mortality.

There has been particular interest in epidemiology in the effects of network structure and topography on the spread of infectious disease. This work reveals the complex interplay between network-structural characteristics and the nature of infectious disease spread through transmission networks, both of which affect model choice and parameterization. With regard to assumptions and models of infection, a key distinction is between the susceptible-infected-removed (SIR) model, which assumes a diminishing pool of infected individuals focusing on time to or probability of infection, and more refined susceptible-infected-susceptible (SIS) models, which allow for backtracking (e.g., one infects a friend who then reinfects the original source after he/she has recovered). This has enormous implications in recurrent-state epidemics and where there is no irreversible spreading due to permanent acquired immunity [16].

Regarding network topology, insights such as the above highlight the contingent role played by network-structure parameters under different infectious disease contexts. Drawing on network epidemiology and related approaches, important concerns include widespread assumptions regarding the operation of preferential attachment and resulting extreme skewness in the degree distribution under different infectious disease contexts, network non-directedness, the presence of loops and cyclicality, clustering and small-world properties, weak ties and the bridging of structural holes, assortativity, homogeneity, and the presence of groups in bipartite contexts [17–24].

One conclusion from this line of work is that one needs to pay careful attention to the topological properties of the networks being parameterized. Increasingly sophisticated analyses show that the relevance of an array of structural and topological network features that we already know are relevant to transmission efficiency depends heavily on the nature of the infectious disease being studied, leading to extensions on foundational network-epidemiological models [25]. One example of research on how the role of network-structural features depend on the nature of the epidemic infectious disease process can be found in work on competing activation mechanisms that operate within the topology of a network. Research shows that the innermost core of a network boosts infectious disease transmission in the context of transient-state epidemics, while in steady-state epidemics there is dual significance in the role played by hubs and the core [26]; see also [27].

The reciprocal effects of epidemic spread on network-structural characteristics, degree topography, and social contact patterns have also affected how network evolution is taken into account and parameterized in simulation models [28]. Research along these lines has focused on the dynamic nature of dynamic real-world networks under the strain of ongoing epidemics, as when taking into account rates of network withdrawal and self-isolation [29–31]. This becomes an even more complex modeling challenge given other social-structural factors that play a role in shaping different nodes’ propensities for withdrawal, isolation, and clustering. This involves factors ranging from the positions individuals occupy within the broader topography of real-world networks (e.g., whether they are brokers, hubs, or already isolates), socio-demographic factors like age, race/ethnicity, income, occupation (e.g., “essential worker” status), and the presence of structural opportunities/constraints on withdrawal, and their pre-existing embeddedness in numerous overlapping social foci or contexts, especially school, the family an household, community voluntary associations, and the workplace [32–34]. Considering the granular details of interpersonal contact (e.g., frequency. volume) and the contexts in which it occurs is critical. An individual’s level of network connectedness – especially network size and levels of contact with others in these everyday settings – are some of the most important factors that contribute to infectious disease susceptibility and, by extension, spread of disease within communities.

Along these lines, epidemiologists have focused increasingly on the effects of *population-level* social-structural factors such as population density, levels of social and material disadvantage, racial/ethnic composition, public transportation systems, vaccination rates, and politically driven policy, among others [35–41]. We expand on these bodies of research by contextualizing the role of network topography – namely, the presence of hubs and heavy degree skewness – on epidemic spread in light of population-level involvement while taking into account a variety of social foci in conjunction with heterogeneity in infection rates during transient-state epidemics that involve burstiness and waves over the long term [22,27,42] We do so by considering the interplay of two key sets of factors: (1) The characteristics of the social networks and contact opportunity structures that exist within communities; and (2) The transmissibility of a given virus within a community, given its infectiousness. We pay particular attention to the possibility that social network topography *conditions* the role that infectiousness plays in the risk of the epidemic spread of disease throughout the community.

### Influence of network hubs and degree skewness on the spread of disease

It is well established that there is considerable heterogeneity with respect to network degree [43,44]. most social networks are characterized by a skewed degree distribution that corresponds to a network with a few highly connected people known as “hubs” and many people who have fewer ties. The same can be said for the distribution of the number of social contacts people have daily – with some people (e.g., grocery store clerks, teachers) having numerous contacts and others having few. Well-connected individuals often play larger roles in spreading diseases like HIV/AIDS, where those occupying bridging positions in sexual networks significantly influenced the epidemic by spreading it into different regions of a community’s sexual network [45,46].

Reflecting the variation among individuals, different populations or communities are characterized by different overarching network structures [47,48]. Studies have suggested that greater heterogeneity in network connectedness, as opposed to the presence of uniformly densely connected or random networks, have contributed to the epidemic spread of the disease at this population level [44,45,49,50]. Recent pandemics like HIV/AIDS and COVID-19 have underscored the importance of understanding the role network degree distribution plays in disease transmission. Research – including analysis of contact tracing data – shows that people with more social connections are more likely to contract and transmit SARS-CoV-2, and that large gatherings facilitate epidemic spread [41,45,51–53]. This insight underpins disease prevention and control strategies like social distancing, community quarantines, and university lockdowns to curb COVID-19 and other pandemics via reducing person-to-person contact [54–61], as involvement in social activities and sustained social contact [62] is crucial for virus transmission, whether through respiratory fluids or surfaces. At the network level, some studies have shown a highly skewed distribution in the transmission network of the COVID-19 transmission network [55,63]. It has been reported that the maximum degree in the transmission network in one wave ranges from 64 to 127, while the average degree in the transmission network is just between 3.8 and 6.4 [64].

Although the spreading network differs from a potential contact network, these results suggest that a few individuals act as hubs in their community networks, with this social network heterogeneity potentially contributing to virus “hot spots” [47,62,65,66]. Despite this, the specific impact of a contact network’s degree distribution on disease spread – and its interaction with transmission rate – remains unclear. This uncertainty is partly due to the lack of empirical data on contact networks, which both necessitates and complicates efforts to accurately model transmission dynamics using simulations.

Additionally, the transmissibility of a pathogen varies widely across virus types and their variants. The initial Alpha variant of SARS-CoV-2, for example, had an estimated basic reproduction number (R0) of around 2.9 [64], while the Delta variant’s R0 is estimated between 3.2 and 6 [67,68]. The Omicron sub-variants exhibit substantially greater transmissibility than earlier lineages, with estimates of the basic reproduction number (R₀) averaging around 9.5 across studies and reaching as high as 24; however, these values vary considerably with local factors such as vaccination coverage, prior infection prevalence, and public-health mitigation measures. [69]. This is on par with more contagious diseases such as measles, which is often pinned at the 12–18 range. [70].

Given the heterogeneity in transmission rates across different types and variants of viruses, it remains uncertain whether social network degree skewness – typically associated with increased epidemic spread in scenarios involving “superspreaders,” or a more skewed social network connectedness (i.e., “degree”) distribution – continues to play a significant role when transmission rates are extremely high. The absence of empirical data on contact networks with sufficient granularity makes it difficult to directly parameterize real-world dynamics. In addition, the inherent complexity of contact-based networks makes it unlikely that an analytical mathematical model with a closed-form solution could adequately capture these processes. For these reasons, simulation modeling provides a suitable framework for getting a sense of the influence of degree skewness under conditions of varying transmissibility.

Seeing these challenges, this paper explores the role of social network structure in disease spread using simulations with SARS-CoV-2-related parameters as useful guideposts. It investigates whether, and to what extent, heterogeneity within individuals’ social networks and contact histories, as described above, shapes the prospect of epidemic spread across variants that have different levels of transmissibility. Disease transmission hinges on social connectedness, so it is vital to understand how the structure of a network in a given community might shape its susceptibility to an epidemic of varying speed. To effectively explore the role of hubs, we conducted simulations informed by empirical data, and isolate the effect of the heterogeneity in individual contacts, while holding constant the dynamics of everyday social contact that occurs within a network.

## Materials and methods

### Social network structure used for simulations

We begin by describing the construction of random social networks used to simulate the epidemic. Specifically, our goal is to generate networks that not only capture the presence or absence of contact between individuals but also incorporate the total duration of contact between each pair of individuals. In this study, network generation is decomposed into two steps. Previous research on real-life social contact data monitored by sensors has shown that social contact data has inter-connected clusters organized by geospatial proximity or shared activities within social foci [71–73]. As a result, we initially construct a random base social network to represent everyday social interactions based on families, schools, workplaces, and daily shopping activities, informed by real-life data (see methodological details in the Appendix). These various venues, termed social foci, serve as focal points where individuals regularly engage in social interactions and daily activities [74]. The construction process generates basic family structures, assigns adults to workplaces and children to schools, and assigns edge weights – approximations of the strength of the contacts – to represent contact levels, assuming uniform random interaction within social foci. The edge weight between two individuals sharing the same foci is inversely proportional to the size of the respective social foci. For example, if a school consists of N students, the edge weight between any two random students in the school is 1/N. If two individuals share more than one foci, the edge weight between them is the accumulation of the weights brought by each of the focus.

This base network provides a structure with a relatively even distribution of contacts. However, real-world social contact data often exhibits inconsistencies in the degree of skewness in contact levels, either measured as the number or the intensity of contacts per individual [33,71–73]. To account for this variation while preserving the general clustering patterns driven by geospatial structures, we introduce heterogeneity in the degree distribution of each simulated base network [33]. Specifically, we add ties with varying contact levels, which can be assortative (leading to a highly skewed distribution with hubs) or disassortative (resulting in a more centralized, uniform distribution). Our aim is to adjust the average total contact level to approximately 50% higher than that of the original base network, introducing realistic variation in contact patterns. This process is referred to as “preferential attachment” in the following text. Friendship edges are added until the target average is reached, with edge weights chosen uniformly at random from the base network, resulting in networks with varying degree distributions but identical total contact levels. While the spreading network of COVID-19 is estimated to be highly skewed, empirical and theoretical work on the degree distributions of individuals’ daily connection networks yields [38,75–77] varied estimates– some suggesting a highly skewed, scale-free distribution [78,79], others assuming more bell-shaped distributions but with an underlying small-world signature [80]. To acknowledge this difference, we manipulate a single skewness parameter to adjust the edges and examine 15 skewness parameters (α), ranging from −4–5, to investigate their impact on degree distribution and its interaction with disease transmissibility. With a skewness parameter greater than 0, the process emulates a preferential attachment mechanism [79], wherein nodes with more connections are more likely to acquire new links. For each base network simulation, we create 15 networks with varying degrees of skewness, maintaining consistent total contact levels across them. A detailed discussion of the selection of skewness parameters, along with summary statistics and their comparison to real-world networks, is provided in the following sections. The process of creating social networks with specific degree distributions is detailed in the Appendix.

### Comparison with existing models

Our model effectively balances the scale-free structure observed in real-world social networks – capturing dyadic interactions, such as friendships that carry infection risks from specific contacts – with broader community-level activities like schooling and shopping, where infection risks arise from shared environments rather than specific individual connections. In constructing the foci network, we incorporate key factors like contact frequency, social-foci size, and exposure duration across settings such as households, workplaces, grocery stores, and schools. This enables our model to more accurately represent variations in transmission potential.

Unlike the Stochastic Block Model which restricts each individual to a single group, our approach allows individuals to participate in multiple groups, reflecting the overlapping social interactions common in real life [81]. While preferential attachment models emphasize high-degree centrality, our method integrates context-sensitive exposure patterns within different social foci [80]. Additionally, classic Erdős-Rényi models [82], which do not take social role differentiation into account, are less suited to capturing the nuanced transmission dynamics that our model achieves by considering both contact intensity and transmission risk.

*Procedure for Generating the Household Network:* To construct a network that meaningfully encapsulates the structures and contexts that characterize daily social life, we employ a social-foci framework as our foundational model. This concept, introduced by Feld [74], identifies various venues – ranging from workplaces and schools to churches, bars, and shopping centers – as focal points where individuals regularly engage in social interactions and daily activities. As an initial step in our network construction, we generate a basic family structure within this broader social-foci framework. To accomplish this, we iterate through individuals within our simulated sample in a sequential manner. At each step, there is an 80% probability that a new individual is marked as part of a household unit, paired with the subsequent individual in the sequence. Once this pairing is established, zero to three subsequent individuals are randomly selected via uniform random sampling to represent their children. After completing the assembly of one household, we reset the procedure and apply the aforementioned steps to generate subsequent households, beginning with the next available individual in the sample.

*Procedure for Generating Social Network Foci*. In addition to families, we integrate individuals into other distinct social foci. For the purpose of this study, we concentrate on three primary social foci: workplaces and grocery stores for adults and schools for children. These social-structural assumptions are not so much intended to imitate the real-life scenario, except that individuals are bound into social foci, wherein much of their contact occurs [75]. As a result, during the foci-network construction, the sizes of these foci and the selection of parameter estimates approximate the density of real cities, but they are not the variable of interests.

To model *workplaces*, we consider the total network size N and generate 18N/1000 workplaces, meaning that there are 18 workplaces for every 1,000 individuals in the network, which is 180 workplaces in our network since N = 10,000. The U.S. population in 2023 was around 335.0 million [83], and, as of 2020, there are 6.1 million employer firms in the United States [84]. This is roughly 18 employer firms per 1000 individuals. According to the United States Bureau of Labor Statistics, the employment rate as of May 2023 is 60.2% [85]. Sixty percent of adults in the network are designated as employed. Each employed adult is then randomly assigned to a workplace, following a power-law distribution with an exponent of −1/2. In particular, for workplace number k (k∈1,2,3,…,180), the probability that an employed adult being assigned to each of the workplace is proportional to $k^{-\text{1}/\text{2}}$.

For *schools*, we posit that one educational institution exists for every 2,500 individuals in the network, since according to National Center for Education Statistics, there are 128961 pre-K-12 schools in the United States, which is an average of one out of every 2600 individuals [86]. Each child is given a 75% probability of being enrolled in a school. According to National Center for Education Statistics [87,88], there are 50.8 million students enrolled in public PK-12 education and 4.7 million students enrolled in private PK-12 education. According to the United States Department of Justice, Office of Juvenile Justice and Delinquency Prevention [89], 72.8 million Americans are between the ages of 0 and 17. This is an enrollment rate of 76.2%. School assignments for children are made through uniform random sampling across the available schools.

For *grocery stores and shopping,* similar to schools, we assume the existence of one grocery store for every 2,500 individuals. According to the United States Department of Agriculture, Economic Research Service [90], there are 115,526 food stores in the United States, which is an average of one out of every 2,900 individuals. Within each household, an adult is randomly selected to undertake grocery shopping responsibilities. It is assumed that this individual frequents the same grocery store, chosen via uniform random sampling from the available options.

*Determining Contact Level per Household and Foci.* Within these foci, we assume individuals to interact with each other uniformly at random, as epidemiologists typically assume of the entire population. The strength of these contact ties, which represents the total duration of contact, is assumed to be inversely proportional to the size of the foci which entails them. To put this assumption in context, for instance, a member in a workplace of six workers has a higher average contact level with every individual co-worker compared to a worker in a workplace of 100.

In addition, people spend much less time on average on grocery shopping each week than on workplaces, families and schools. We assume that the chance of being infected by disease is lower for each possible contact happening in grocery stores by a factor of *k*. Here we set k as 1/40, which comes from the assumption that an individual works for 40 hours on average per week, while this individual goes to the grocery store for one hour in total weekly.

Mathematically, the contact level $w_{i,j}$ between individual *i* and *j* is the sum of the contacts that they have in each of their shared foci g. That is,


$$w_{i,j}=\sum {\mathrm{\backslash nolimits}}_{g}\frac{\delta_{i,g}\delta_{j,g}}{\left|g\right|}\cdot k^{\delta_{\left(g=grocery\right)}},$$

$\delta_{i,g}=\text{1}$ when individual *i* belongs to the foci g and $\delta_{i,g}=\text{0}$ if otherwise. |g| is the number of individuals included in foci g. $\delta_{\left(g=grocery\right)}=\text{1}$ when the foci is a grocery store, under which the chance of infected per unit contact is discounted by *k*.

### Generating a degree-skewed social network given a base network

Based on the foci network, we now describe the key independent structural variable, which generates networks with varying degrees of skewness. We introduce this variable by adding friendship ties, generated independently from the social foci structure of our imaginary city. The variation comes in how assortative or disassortative these network ties are. In particular, we start by setting a target average total contact at 50% above the base network. We define total contact as $\sum_{e\in E}w(e)$, where E denotes the set of all edges in the graph, and w(e) is the weight of the edge.

We then add edges sequentially until this target is reached. We choose the first node of an edge uniformly at random from all nodes, and choose the partner node, say *i*, with probability proportional to $d(i{)}^{\alpha }$, where α encodes the importance of degree in deciding the partner. The weight of the edge is chosen uniformly at random from the existing edges in the base network, and edges are added until the target average is reached. When α= 0, partners are chosen uniformly at random. When α> 0, partners are chosen with higher probability if they have a higher degree. When α< 0, partners are chosen with lower probability if they have higher degree. For this simulation, we used the following skewness parameters: −4, −2,-1, 0 (meaning no skewness compared to the base network), 1, 1.5, 2, 2.5, 3, 3.2, 3.5, 3.8, 4, 4.5, 5, which empirically corresponds to Gini co-efficient of 0.10, 0.12, 0.14, 0.16, 0.18, 0.19, 0.20, 0.22, 0.23, 0.25, 0.26, 0.28, 0.29 with relatively even spacing between them than the range of the alpha parameters have suggested. Furthermore, prior research on interpersonal social contact reports largely different levels of variation in the contact durations, and squared coefficients of variation (SCV) for contact duration between 0.2 and 12.6 [71–73], and our simulation extends this range by generating SCV values from 0.03 to 121.37, thereby capturing and expanding the observed variability in real-world networks. Networks generated in this way have different degree distributions, but the same total contact.

**Fig 1:**
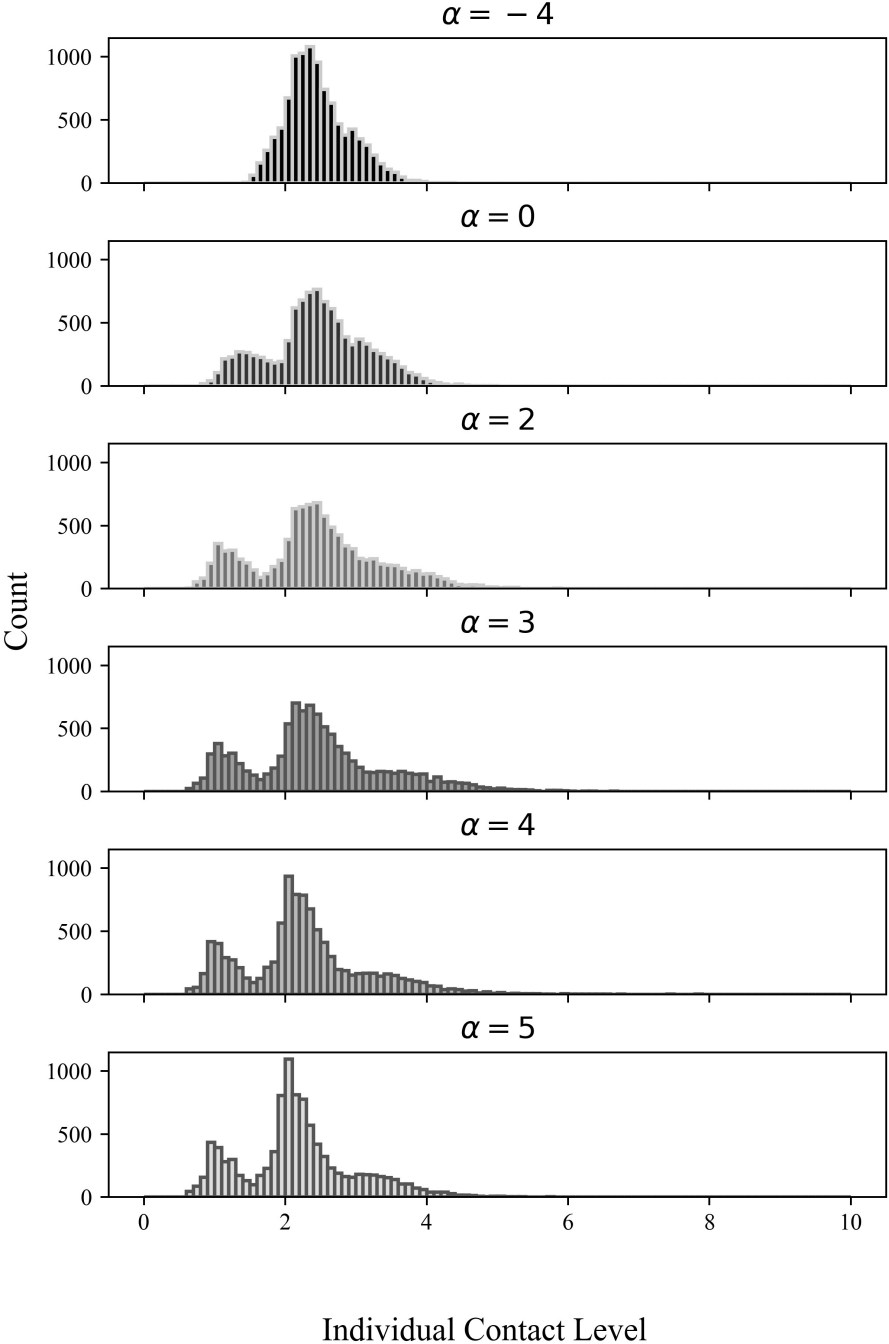
The simulated impact of α on the skewness of a social context-level distribution (N = 10,000) This figure is based on a comparison of the aggregate weighted degree distributions among individuals in a sample of the final networks that are based on one of the randomly-generated base foci-networks we examine in this paper. Alpha parameterizes the amount of assortativity in the ad-hoc friendships which are added to the same base network of schools, work, family, and grocery social circles. Total contact in the society is equal across networks, and thus the mean level of contact per person is constant. The long right tails are omitted for better visualization.

To illustrate the effectiveness of our process on altering the network degree distribution, we sample a social foci base network comprising 10,000 individuals. The resulting base network has an initial total contact level (i.e., the sum of the existing edge weights) of 8,220.5. After each preferential attachment process with varying skewness parameters, every final network reaches a final contact level of 12331. The degree distributions of the exemplar final networks could be found in Fig 1, and the summary statistics of the degree distribution can be seen in Table 1. In Fig 1, for a network adjusted with a skewness parameter of −4, the individual with the maximum weighted degree has a contact level of 4.24, while the individual with the minimum weighted degree has a contact level of 1.46. Conversely, in a network adjusted with a skewness parameter of 4, the maximum individual contact level skyrockets to 2722.23, while the minimum drops to 0.64 (right tail of the distribution that has skewness equal to 4 is omitted from Fig 1 due to space restraints.).

**Table 1:** Descriptive statistics illustrating the impact of α on the skewness of a social context-level distribution, using different α on the same base network.

*α*	Min	Max	Mean	Median	Gini-coefficient
−4	1.46	4.24	2.47	2.39	0.10
0	0.80	5.18	2.47	2.45	0.16
2	0.68	6.34	2.47	2.43	0.20
3	0.65	9.36	2.47	2.37	0.22
4	0.65	1146.76	2.47	2.25	0.26
5	0.64	2272.23	2.47	2.13	0.29

**Fig 2:**
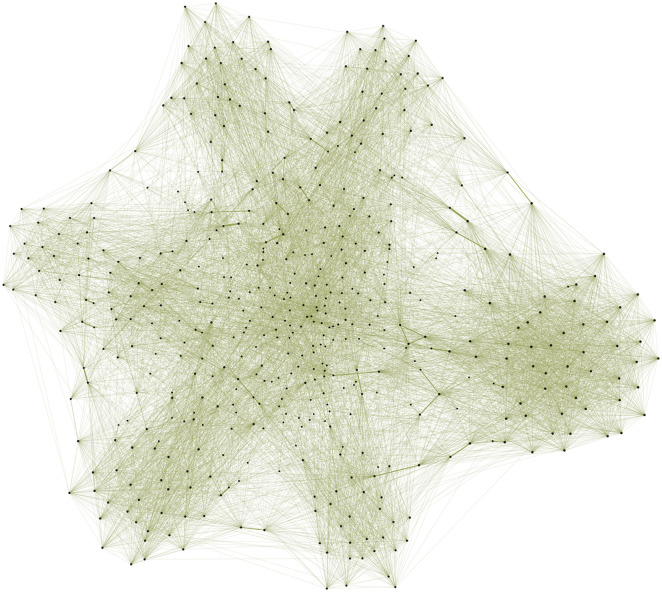
Focused illustration of a small part (500 nodes) of the social network with the presence of hubs/skewness in the community infection simulation. The thickness of network edges represents the amount of total contact between individuals, and the size of the node is the contact level of the individual. To generate this graph, we choose the top 10 individuals with the highest contact level and then randomly sample 490 other individuals in the graph. We can see that the focal person in the middle is very well-connected, while other individuals are significantly less well-connected, which exemplifies a larger trend across this degree-agnostic network.

**Fig 3:**
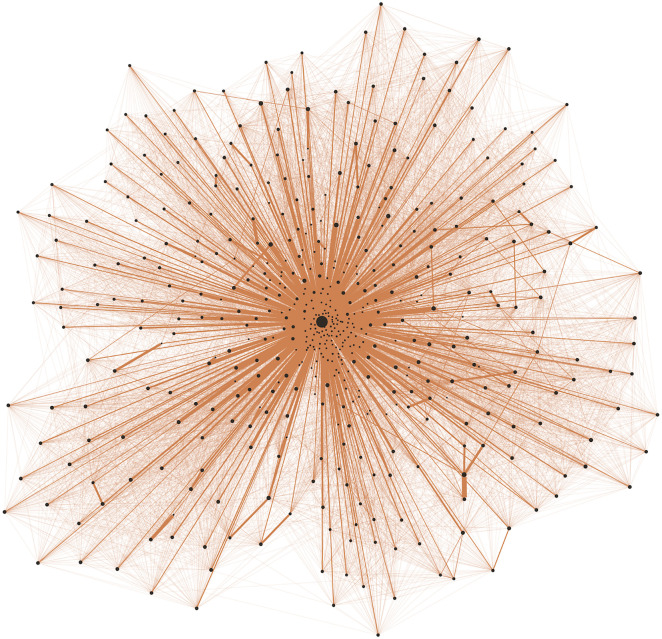
Focused illustration of a small part (500 nodes) of the social network with the lowest prevalence of hubs/skewness in the community infection simulation. The thickness of network edges represents the amount of total contact between individuals, and the size of the node is the contact level of the individual. To generate this graph, we choose the top 10 individuals with the highest contact level and then randomly sample 490 other individuals in the graph. We can see that every node seems to be evenly connected, which exemplifies a larger trend across this degree-agnostic friendship network.

In Figs 2 and 3, we compare two randomly generated networks based on the same base network with 10,000 individuals: one with a highly skewed degree distribution (α=4, Fig 2) and the other with a more centered distribution (α= −4, Fig 3). To facilitate better visualization in both figures, we only plot 500 nodes, selecting the 10 with the highest node degree in the graph and randomly choosing the other 490. As shown in the visualizations, the highly skewed network (Fig 2) features a single hub with connections to most of the other nodes, indicating a centralized structure. In contrast, the disassortative network (Fig 3) lacks a node with an extremely high degree, suggesting a more evenly distributed network without dominant hubs.

### Simulating epidemic spread and population-level susceptibility

We simulate disease spread using a continuous-time, network-based stochastic SEIR (Susceptible–Exposed–Infectious–Recovered) model [91] implemented via the Gillespie algorithm, as provided by the EoN (Epidemics on Networks) Python package [92]. Different from traditional approaches that relies on global-level density or local-level frequency, in this network-based approach, each susceptible individual’s transition from Susceptible (S) to Exposed (E) depends on the presence of local infectious neighbors, with each contact considered independently. Specifically, for a susceptible individual i and an infected neighbor j at any time of the simulation, the rate of exposure for i from j, $\lambda_{j\to i}$, is:


$$\lambda_{j\to i}=\beta_{se}*w_{ij}$$

where $\beta_{se}$ is the transmission rate per unit contact, and $w_{ij}$ is the edge weight or concretely contact level between the two nodes. In cases that a susceptible node has several infected neighbors, the infection from each neighbor is independently considered.

In contrast, transitions from Exposed (E) to Infectious (I) and from Infectious (I) to Recovered (R) occur at constant rates, independent of network structure. All transitions are modeled as exponentially distributed waiting times, a standard assumption in epidemic modeling due to the memoryless property of the exponential distribution [93]. While we recognize that real-world transition times may not strictly follow an exponential distribution, we leave this refinement to future work. To maintain clarity and focus, we provide detailed parameter settings in this section. And leave simulation replication protocols at the beginning of the results section.

To approximate the early spread of COVID-19, we initialize each simulation with 5% of individuals randomly assigned to the infectious state. We systematically vary the disease’s infectiousness by adjusting $\beta_{se}$, rate of transmission per contact in our simulation, which is inversely related to the expected time to infection. This adjustment allows us to model a wide range of real-world conditions, from strict social distancing to minimal intervention, as well as inherent differences in disease transmissibility. For a given unit contact level, this rate is scaled from 0.01 to 0.5, corresponding to expected infection times per contact ranging from 100 days to 2 days. To put this in more context, in a hypothetical family of three members who do not share workplaces, shopping routines, or expand contacts through preferential attachment and given $\beta_{se}$ equal to 0.1, the rate of infection from an infectious to a susceptible member is 1/3 (contact level) * 0.1 ($\beta_{\text{se}}$)= 1/30, resulting in an expected time to transmission of 30 days. If two members of the family are infected and connected only through the family foci link with the remaining susceptible member, the expected time to transmission to the remaining member from the family foci is approximately 15 days with a median of around 10.4 days.

Studies have shown that the average incubation period for COVID-19 variants ranges from 5.0 days for the Alpha variant to 3.42 for the Omicron Variant [94]. To cover this variation, we set our exposed to infection parameter, $\beta_{ei}$, to 1/2 or 1/5, corresponding to an incubation period of 2 days and 5 days. To account for the recovery rate, we set $\beta_{ir}$ at 0.1, an expected time to recovery at 10 days, which lies in the middle of recovery durations listed by George et al. [95].

For COVID-19, there is conflicting evidence on the rates of re-susceptibility ($\beta_{rs}$). Guedes et al. [96] measured reinfections amongst healthcare workers March 2020 to March 2022, and found that reinfection was present in only 5% of cases, with an average reinfection interval of 429 days. These findings motivate our use of an SEIR model in the main simulation. However, emerging evidence on reinfection dynamics prompts us to conduct robustness checks using an SEIRS model that incorporates reinfection probabilities. For instance, Hu and Geng [97] used a heterogeneous SIRS model to estimate clusters of parameters across the United States in April 2020, finding a 95% highest probability range of the rate of re-infection $\beta_{rs}$ of $\left(\text{1}.\text{1}\text{8}\text{1}\times {\text{1}\text{0}}^{-\text{7}},\text{0}.\text{0}\text{0}\text{4}\text{7}\right)$. The upper value represents an average reinfection time of 213 days. On the other hand, Nguyen et. al [98] analyzed 17 studies and reported an average re-infection interval of 178.9 days, while the variation between studies is large. In addition, they mentioned that the reinfection rates are higher with the Omicron Variants, which partially explains the inconsistencies across studies. In our study, focusing on the total number of infections in a single outbreak (defined as the sudden occurrence of a greater-than-expected number of cases of the disease or variant in question relative to baseline within a given time frame), we set our reinfection rate to zero to clearly observe the effect. However, to assess the peak of the outbreak (i.e., the maximum number of individuals being infected at the same time), we conducted robustness checks with two other reinfection rates: one akin to influenza, resulting in reinfection every 60 days on average, and the other closer to the findings of Hu and Geng [97], with reinfection every 200 days. These checks help validate our findings on the maximum daily number of infections.

All parameters tested in the simulation are documented in Table 2. Overall, the parameters chosen in this study are meant to constitute an illustration of a more general phenomenon. Determining what extent our findings generalize across every choice of parameters constitutes important future work.

**Table 2:** Description and notation for parameters – including ranges employed and example descriptions of how these translate to our simulation of disease spread.

Parameter	Description	Value
$\beta_{se}$	The rate at which the infected expose their contact partners	[0.01, 0.02, 0.04, 0.05, 0.06, 0.07, 0.08, 0.09, 0.1, 0.2, 0.15, 0.25, 0.33, 0.5] 0.10 corresponds to once in 10 days, per contact weight with infected individuals
$\beta_{ei}$	The rate at which exposed individuals become infectious	1/2, 1/5 (e.g., 1/5 corresponds to once in 5 days, on average)
$\beta_{ir}$	The rate at which infected individuals recover from infection	1/10 (corresponds to once in 10 days, on average)
$\beta_{rs}$	The rate at which recovered individuals become susceptible again	[0, 1/60, 1/200] (e.g., 0 corresponds to never, 1/60 corresponds to once in 60 days on average)
${inf}_{0}$	At the start of the simulation, a constant percentage of random individuals are infected	5%
α	The skewness of the network and the presence of hubs	[-4, -2,-1, 0, 1, 1.5, 2, 2.5, 3, 3.2, 3.5, 3.8,4, 4.5, 5] 0 means no skewness comparing to the base network, while a positive value means the network degree distribution is more skewed, indicating the presence of hubs

## Results

Our simulation models offer novel insights into the relationship between the degree distribution of social networks and the spread of infectious diseases. For this study, we kept $\beta_{ir}$ fixed at 0.1 and $\beta_{rs}$ at 0, and randomly create 300 base networks with 10,000 individuals for each unique set of epidemic-related parameters (i.e., $\beta_{se}$, $\beta_{ei}$, $\beta_{ir}$ and ${\text{inf}}_{\text{0}}$). On each base network, we randomly generated 12 final networks via the preferential attachment process where each of them corresponds to one skewness parameter. This within-design ensures that comparisons across skewness levels are made on the same base foci network realization, thereby reducing structural noise and improving the validity of our inference. We run one simulation on each of the final network generated, which results in 300 simulations for each skewness and epidemic parameter combinations. We ran each simulation until the system reaches equilibrium: in an SEIR model, this means all individuals are recovered and no more individuals are infected.

**Fig 4:**
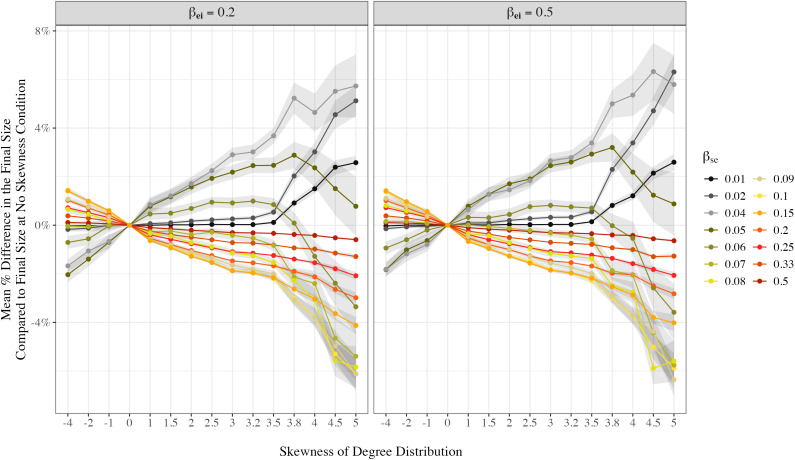
Impact of the presence of hubs/skewness in social contact networks on final epidemic size in communities. Fig 4 demonstrates the influence of degree-skewness, and thus the prevalence of hubs, in casual social contact networks on the total number of uninfected individuals. This figure takes into account the varied infectiousness of the disease ($\beta_{se}$), network skewness, and exposure to infectious rate ($\beta_{ei}$). In these simulations, $\beta_{ir}=\text{0}.\text{1}$, $\beta_{rs}=\text{0}$ for each parameter combination. The y-axis presents the average numerical difference in final sizes between a foci-network with degree-skewness and one with zero skewness given the same base network. The 95% confidence interval is calculated based on the mean absolute difference of final size between the zero-skewness baseline and each alternative skewness condition, expressed as a percentage. For each contagion rate, 300 base foci-networks were simulated. These base networks were then modified by varying preferential attachment rules, guided by different skewness parameters. Each modified network comprises 10,000 individuals.

We first explore final size of an outbreak: The proportion of people having been infected at the end of a single outbreak given varied disease infectiousness ($\beta_{se}$), network degree skewness, and exposure to infection rate, using an SEIR model. Fig 4 shows a key metric concerning the final size of a single outbreak of simulated epidemic: The numerical difference of final size relative to the zero-skewness network, given $\beta_{ei}=\;\text{0}.\text{2}$.

In our simulations, while keeping other transmission parameters stable, we found that higher contagion rates ($\beta_{se}\geq \text{0}.\text{0}\text{8}$), networks that have a more skewed degree distribution than the less-skewed ones tend to result in fewer individuals infected in total, assuming the overall number of contacts within the population remains the same. Conversely, at lower contagion rates ($\beta_{se}=\;\text{0}.\text{0}\text{1},\;\text{0}.\text{0}\text{2},\;\text{0}.\text{0}\text{5}$), more individuals in total are infected in networks with higher degree skewness than those with lower degree skewness. For instance, with a contagion rate of $\beta_{se}=\;\text{0}.\text{0}\text{2}$ and $\beta_{ei}=\;\text{0}.\text{2}$, high skewness exposes more individuals to risk compared to a zero-skewness condition: foci-networks with α= 5 protects 512 (5.1% of the total population) individuals fewer on average than its zero-skewness counterparts built on the same base networks.

As Fig 4 illustrates, a phase transition appears to occur somewhere around *ß*_se_ = 0.05 and *ß*_se_ = 0.06. In this region we see the inflection point, or contagion threshold, where increasingly extreme skewness beyond this results in a reversal in an upward trend in final size to such an extent that final size is actually lower than it is when there is no skewness (i.e., dipping below mean difference of 0), given the skewness ranges we included in this simulation. It is the level of contagion where, as we move beyond that, out of lulls and into periods of increasingly widespread epidemic activity, hubs appear to begin to lose their power to drive epidemic spread. At $\beta_{se}=\;\text{0}.\text{1}$ and $\beta_{ei}=\;\text{0}.\text{2}$, high skewness results in greater protection than its zero-skewness counterpart: foci-networks with α= 5 protects 611 (6.1% of the total population) individuals more on average than its zero-skewness counterparts built on the same base networks.

In addition, the effect of degree skewness is strongest when the disease is neither extremely contagious nor very mild. This could be found by comparing the absolute difference between the number of total infected individuals in a highly-skewed network versus a zero-skewness network (α= 0) on different contagion rates (Fig 4). Again, at $\beta_{ei}=\;\text{0}.\text{2}$, a highly skewed network (α= 5) protects on average 6.1% more people in the population from being infected given than a no preferential mechanism (α= 0) network with a relatively high contagion rate of $\beta_{se}=\;\text{0}.\text{1}$, while it only protects an average of 0.6% more individuals than a zero-skewness network when the contagion rate is even higher at $\beta_{se}=\text{0}.\text{5}$. Table 3 details the proportion of the total population that have been infected at the conclusion of the simulated epidemic without re-infection. While the interpretation uses simulations with $\beta_{ei}=\;\text{0}.\text{2}$ (Table 3), conclusions are similar at $\beta_{ei}=\;\text{0}.\text{5}$ (Table 4).

**Table 3:** Average final size (i.e., proportion of total population infected at the conclusion of an epidemic), by the infectiousness of the disease ($\beta_{se}$) and the presence of hubs (network skewness) in this simulation, $\beta_{ei}$ = 0.2 (exposure to infection rate). $\beta_{ir}=0.1$, $\beta_{rs}\;=\;\text{0}$. For each parameter combination, 300 simulations were run.

		Skewness Parameter α														
		−4	−2	−1	0	1	1.5	2	2.5	3	3.2	3.5	3.8	4	4.5	5
Disease Infectiousness $\beta_{se}$	0.01	6.5% (±0.02%)	6.5% (±0.02%	6.5% (±0.02%)	6.5% (±0.02%)	6.5% (±0.02%)	6.5% (±0.02%)	6.5% (±0.02%)	6.5% (±0.02%)	6.5% (±0.02%)	6.5% (±0.02%)	6.6% (±0.04%)	7.4% (±0.15%)	8.0% (±0.19%)	8.9% (±0.29%)	9.1% (±0.28%)
	0.02	9.0% (±0.05%)	9.0% (±0.05%)	9.1% (±0.05%)	9.1% (±0.05%)	9.2% (±0.05%)	9.2% (±0.05%)	9.3% (±0.05%)	9.4% (±0.05%)	9.4% (±0.05%)	9.4% (±0.06%)	9.7% (±0.09%)	11.2% (±0.26%)	12.2% (±0.39%)	13.7% (±0.58%)	14.3% (±0.63%)
	0.04	24.0% (±0.18%)	24.6% (±0.21%)	25.0% (±0.21%)	25.7% (±0.20%)	26.5% (±0.21%)	26.9% (±0.21%)	27.4% (±0.19%)	27.9% (±0.21%)	28.6% (±0.21%)	28.7% (±0.22%)	29.4% (±0.25%)	30.9% (±0.60%)	30.3% (±0.76%)	31.2% (±1.06%)	31.4% (±1.29%)
	0.05	40.1% (±0.23%)	40.7% (±0.24%)	41.4% (±0.20%)	42.0% (±0.22%)	42.8% (±0.22%)	43.2% (±0.20%)	43.6% (±0.20%)	44.0% (±0.21%)	44.2% (±0.19%)	44.5% (±0.20%)	44.9% (±0.24%)	44.5% (±0.51%)	44.4% (±0.76%)	43.6% (±1.08%)	42.8% (±1.21%)
	0.06	55.6% (±0.20%)	55.8% (±0.18%)	56.3% (±0.18%)	56.4% (±0.17%)	56.8% (±0.17%)	56.8% (±0.16%)	57.0% (±0.16%)	57.3% (±0.15%)	57.3% (±0.15%)	57.3% (±0.16%)	57.2% (±0.21%)	56.4% (±0.41%)	55.1% (±0.61%)	54.0% (±0.92%)	53.0% (±1.14%)
	0.07	67.3% (±0.14%)	67.3% (±0.14%)	67.3% (±0.14%)	67.3% (±0.15%)	67.1% (±0.14%)	67.1% (±0.13%)	66.9% (±0.13%)	67.0% (±0.12%)	66.9% (±0.13%)	66.8% (±0.12%)	66.5% (±0.17%)	65.2% (±0.34%)	64.9% (±0.52%)	62.7% (±0.80%)	61.9% (±0.97%)
	0.08	75.5% (±0.11%)	75.3% (±0.11%)	75.0% (±0.11%)	74.9% (±0.10%)	74.5% (±0.11%)	74.4% (±0.11%)	74.1% (±0.10%)	74.0% (±0.11%)	73.7% (±0.11%)	73.6% (±0.10%)	73.3% (±0.13%)	72.6% (±0.30%)	71.8% (±0.46%)	69.3% (±0.64%)	69.0% (±0.86%)
	0.09	81.5% (±0.09%)	81.2% (±0.09%)	80.8% (±0.09%)	80.4% (±0.09%)	79.9% (±0.09%)	79.6% (±0.09%)	79.3% (±0.09%)	79.1% (±0.09%)	78.8% (±0.08%)	78.6% (±0.09%)	78.4% (±0.12%)	77.4% (±0.23%)	76.6% (±0.39%)	75.5% (±0.62%)	74.3% (±0.70%)
	0.1	85.9% (±0.08%)	85.4% (±0.08%)	85.0% (±0.07%)	84.4% (±0.08%)	83.9% (±0.07%)	83.6% (±0.08%)	83.3% (±0.08%)	82.9% (±0.07%)	82.6% (±0.08%)	82.5% (±0.07%)	82.2% (±0.09%)	81.4% (±0.21%)	80.6% (±0.32%)	79.1% (±0.49%)	78.3% (±0.62%)
	0.15	95.6% (±0.03%)	95.2% (±0.04%)	94.8% (±0.04%)	94.2% (±0.04%)	93.6% (±0.04%)	93.3% (±0.04%)	92.9% (±0.04%)	92.7% (±0.04%)	92.3% (±0.04%)	92.2% (±0.04%)	92.0% (±0.05%)	91.6% (±0.12%)	91.2% (±0.16%)	90.6% (±0.27%)	90.1% (±0.34%)
	0.2	98.4% (±0.02%)	98.1% (±0.02%	97.8% (±0.02%)	97.4% (±0.02%)	96.8% (±0.03%)	96.6% (±0.03%)	96.3% (±0.03%)	96.1% (±0.03%)	95.9% (±0.03%)	95.8% (±0.03%)	95.7% (±0.03%)	95.5% (±0.07%)	95.3% (±0.12%)	94.7% (±0.18%)	94.4% (±0.22%)
	0.25	99.4% (±0.01%)	99.2% (±0.01%)	99.0% (±0.01%)	98.6% (±0.01%)	98.2% (±0.02%)	98.1% (±0.02%)	97.9% (±0.02%)	97.7% (±0.02%)	97.6% (±0.02%)	97.5% (±0.02%)	97.4% (±0.02%)	97.2% (±0.05%)	97.1% (±0.07%)	96.9% (±0.12%	96.6% (±0.16%
	0.33	99.8% (±0.00%)	99.7% (±0.01%)	99.6% (±0.01%)	99.4% (±0.01%)	99.2% (±0.01%)	99.1% (±0.01%)	99.0% (±0.01%)	98.8% (±0.01%)	98.7% (±0.01%)	98.7% (±0.01%)	98.6% (±0.02%)	98.5% (±0.03%)	98.5% (±0.05%)	98.3% (±0.08%)	98.1% (±0.10%)
	0.5	100.0% (±0.00%)	100.0% (±0.00%)	99.9% (±0.00%)	99.9% (±0.00%)	99.8% (±0.01%)	99.7% (±0.01%)	99.7% (±0.01%)	99.6% (±0.01%)	99.6% (±0.01%)	99.6% (±0.01%)	99.5% (±0.01%)	99.5% (±0.02%)	99.5% (±0.02%)	99.3% (±0.04%)	99.3% (±0.05%)

**Table 4:** Average final size (i.e., proportion of total population infected at the conclusion of an epidemic), by the infectiousness of the disease ($\beta_{se}$) and the presence of hubs (network skewness) in this simulation, $\beta_{ei}$ = 0.5 (exposure to infection rate). $\beta_{ir}=0.1$, $\beta_{rs}\;=\;\text{0}$. For each parameter combination, 300 simulations were run.

		Skewness Parameter α														
		−4	−2	−1	0	1	1.5	2	2.5	3	3.2	3.5	3.8	4	4.5	5
Disease Infectiousness $\beta_{se}$	0.01	6.5% (±0.02%)	6.5% (±0.02%)	6.5% (±0.02%)	6.5% (±0.02%)	6.5% (±0.02%)	6.5% (±0.02%)	6.5% (±0.02%)	6.5% (±0.02%)	6.5% (±0.02%)	6.5% (±0.02%)	6.7% (±0.05%)	7.3% (±0.13%)	7.7% (±0.18%)	8.7% (±0.26%)	9.1% (±0.34%)
	0.02	9.0% (±0.05%)	9.0% (±0.05%)	9.1% (±0.05%)	9.1% (±0.05%)	9.2% (±0.05%)	9.2% (±0.05%)	9.3% (±0.05%)	9.4% (±0.05%)	9.4% (±0.06%)	9.4% (±0.06%)	9.7% (±0.8%)	11.4% (±0.28%)	12.5% (±0.39%)	13.8% (±0.57%)	15.4% (±0.72%)
	0.04	24.0% (±0.20%)	24.6% (±0.21%)	25.0% (±0.18%)	25.7% (±0.22%)	26.5% (±0.20%)	27.1% (±0.22%)	27.3% (±0.21%)	27.7% (±0.21%)	28.5% (±0.21%)	28.6% (±0.23%)	29.2% (±0.26%)	30.8% (±0.54%)	31.2% (±0.84%)	32.2% (±1.17%)	31.6% (±1.20%)
	0.05	40.0% (±0.23%)	40.9% (±0.22%)	41.2% (±0.22%)	41.9% (±0.20%)	42.6% (±0.23%)	43.1% (±0.20%)	43.6% (±0.22%)	43.8% (±0.20%)	44.3% (±0.20%)	44.5% (±0.20%)	44.8% (±0.26%)	45.1% (±0.54%)	44.1% (±0.74%)	43.1% (±1.08%)	42.7% (±1.25%)
	0.06	55.6% (±0.19%)	55.9% (±0.18%)	56.3% (±0.17%)	56.5% (±0.18%)	56.9% (±0.17%)	56.8% (±0.17%)	57.0% (±0.16%)	57.3% (±0.16%)	57.3% (±0.16%)	57.3% (±0.15%)	57.2% (±0.20%)	56.5% (±0.45%)	56.0% (±0.65%)	53.9% (±0.91%)	52.9% (±1.13%)
	0.07	67.3% (±0.15%)	67.3% (±0.14%)	67.2% (±0.14%)	67.1% (±0.13%)	67.1% (±0.13%)	67.1% (±0.13%)	67.0% (±0.12%)	66.9% (±0.12%)	66.8% (±0.12%)	66.7% (±0.13%)	66.6% (±0.14%)	65.3% (±0.37%)	65.1% (±0.52%)	62.7% (±0.74%)	61.4% (±0.99%)
	0.08	75.6% (±0.12%)	75.4% (±0.11%)	75.2% (±0.11%)	74.8% (±0.11%)	74.5% (±0.10%)	74.4% (±0.10%)	74.1% (±0.10%)	73.9% (±0.10%)	73.7% (±0.10%)	73.5% (±0.10%)	73.4% (±0.13%)	72.3% (±0.33%)	72.0% (±0.41%)	68.9% (±0.65%)	69.2% (±0.85%)
	0.09	81.5% (±0.09%)	81.2% (±0.09%)	80.8% (±0.09%)	80.4% (±0.09%)	79.9% (±0.08%)	79.6% (±0.08%)	79.4% (±0.09%)	79.1% (±0.09%)	78.8% (±0.10%)	78.6% (±0.09%)	78.4% (±0.11%)	77.5% (±0.24%)	76.7% (±0.37%)	76.0% (±0.61%)	74.0% (±0.68%)
	0.1	85.8% (±0.08%)	85.3% (±0.07%)	85.0% (±0.07%)	84.5% (±0.08%)	83.9% (±0.08%)	83.5% (±0.07%)	83.2% (±0.07%)	83.0% (±0.08%)	82.6% (±0.07%)	82.5% (±0.07%)	82.2% (±0.10%)	81.5% (±0.23%)	80.7% (±0.31%)	79.4% (±0.49%)	78.6% (±0.63%)
	0.15	95.6% (±0.03%)	95.2% (±0.03%)	94.8% (±0.04%)	94.2% (±0.04%)	93.5% (±0.04%)	93.3% (±0.04%)	92.9% (±0.04%)	92.7% (±0.04%)	92.4% (±0.04%)	92.2% (±0.04%)	92.0% (±0.05%)	91.7% (±0.12%)	91.3% (±0.18%)	90.4% (±0.27%)	90.2% (±0.34%)
	0.2	98.4% (±0.02%)	98.1% (±0.02%)	97.8% (±0.02%)	97.4% (±0.02%)	96.9% (±0.03%)	96.6% (±0.02%)	96.4% (±0.03%)	96.1% (±0.03%)	95.9% (±0.03%)	95.8% (±0.03%)	95.7% (±0.03%)	95.4% (±0.07%)	95.3% (±0.11%)	94.9% (±0.18%)	94.6% (±0.22%)
	0.25	99.4% (±0.01%)	99.2% (±0.01%)	99.0% (±0.01%)	98.6% (±0.01%)	98.3% (±0.02%)	98.0% (±0.02%)	97.9% (±0.02%)	97.7% (±0.02%)	97.5% (±0.02%)	97.5% (±0.02%)	97.4% (±0.03%)	97.3% (±0.05%)	97.1% (±0.08%)	96.8% (±0.12%)	96.6% (±0.15%)
	0.33	99.8% (±0.01%)	99.7% (±0.01%)	99.6% (±0.01%)	99.4% (±0.01%)	99.2% (±0.01%)	99.1% (±0.01%)	98.9% (±0.01%)	98.8% (±0.01%)	98.7% (±0.01%)	98.7% (±0.01%)	98.6% (±0.02%)	98.5% (±0.03%)	98.4% (±0.05%)	98.1% (±0.08%)	98.2% (±0.10%)
	0.5	100.0% (±0.00%)	100.0% (±0.00%)	99.9% (±0.00%)	99.9% (±0.00%)	99.8% (±0.01%)	99.7% (±0.01%)	99.7% (±0.01%)	99.6% (±0.01%)	99.6% (±0.01%)	99.6% (±0.01%)	99.5% (±0.01%)	99.5% (±0.02%)	99.5% (±0.02%)	99.3% (±0.04%)	99.2% (±0.05%)

In substantive terms, these findings challenge conventional wisdom about the role of hubs – or highly connected individuals – in disease transmission. Specifically, when the total level of contact within a population is held constant, though hubs themselves are connected to a greater number of individuals, their presence also results in a median individual who is less well-connected, which may form a demographic that is potentially resistant to infection. Our results

Indicate that infectiousness of the disease influences whether a skewed network structure protects the community or exposes the community to higher risk, highlighting the importance of tailored public health strategies.

**Fig 5:**
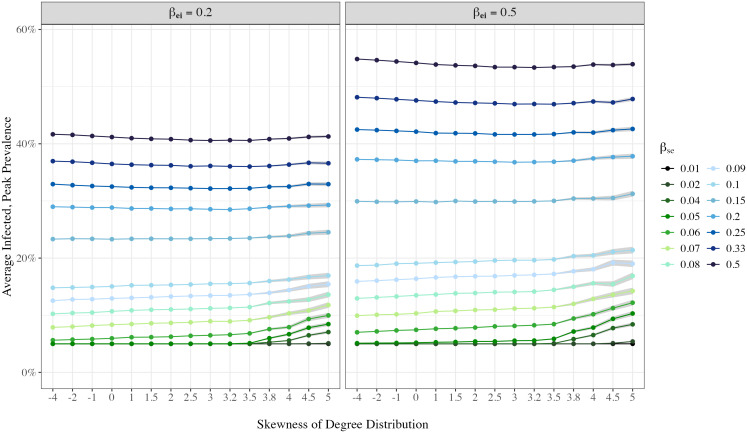
Impact of the presence of hubs/skewness in casual contact networks on peak prevalence of infection in communities by disease infectiousness (*ß*_se_) Estimates are varied by the infectiousness of the disease ($\beta_{se}$), network skewness and exposure to infectious rate ($\beta_{ei}$). In this simulation, $\beta_{ir}=\text{0}.\text{1}$, $\beta_{rs}=\text{0}$. For each parameter combination, 300 simulations were run. 95% confidence bands were drawn.

While the overall number of cases during an outbreak is important, the number of individuals being infected at the peak of the outbreak offers a unique and critical insight. This number serves as a proxy for the peak demand on healthcare resources, such as hospital beds and medical supplies. Fig 5 presents the proportion of infected individuals at the peak of infection, $\beta_{ei}=\text{0}.\text{2},\;\text{0}.\text{5}$. When we keep the infectiousness of the disease steady, networks with more pronounced hub structures –indicated by higher skewness – are linked to a surge in peak infections, particularly when the disease has moderate infectiousness ($\beta_{se}=\text{0}.\text{0}\text{5}$ to 0.1) In addition to this pattern, we observed that the peak prevalence is consistent at 5% for some parameter combination, equal to the initial infection level specified in Table 1. While this may suggest limited spread, it does not mean that an outbreak did not occur: in some parameter settings, as large as around 43% of the population is ultimately infected, but the maximum number of people being infected never exceeds 5%. These scenarios imply a slow-moving contagion that, despite reaching a large portion of the population over time, avoids sharp surges that would place acute pressure on the healthcare system. However, when a disease is highly infectious, the impact of network skewness appears to diminish: there is not many substantive differences by network skewness in the maximum number of individuals being infected at the same time. Corresponding point estimates are found in Tables 5 and 6. To validate these findings, robustness tests were conducted using SEIRS models with $\beta_{rs}=\frac{\text{1}}{\text{6}\text{0}},\frac{\text{1}}{\text{2}\text{0}\text{0}}$. These tests, simulating for 500 days with 300 replications for each parameter set, yield similar conclusion and are detailed in S1 Fig.

**Table 5:** Average infected peak prevalence, varied by the infectiousness of the disease ($\beta_{se}$) and presence of hubs (network skewness) in this simulation, $\beta_{ei}$ = 0.2 (exposure to infection rate). $\beta_{ir}=0.1$, $\beta_{rs}\;=\;0$. For each parameter combination, 300 simulations were run.

		Skewness Parameter α														
		−4	−2	−1	0	1	1.5	2	2.5	3	3.2	3.5	3.8	4	4.5	5
Disease Infectiousness $\beta_{se}$	0.01	5.0% (±0.00%)	5.0% (±0.00%)	5.0% (±0.00%)	5.0% (±0.00%)	5.0% (±0.00%)	5.0% (±0.00%)	5.0% (±0.00%)	5.0% (±0.00%)	5.0% (±0.00%)	5.0% (±0.00%)	5.0% (±0.00%)	5.0% (±0.00%)	5.0% (±0.00%)	5.0% (±0.00%)	5.0% (±0.00%)
	0.02	5.0% (±0.00%)	5.0% (±0.00%)	5.0% (±0.00%)	5.0% (±0.00%)	5.0% (±0.00%)	5.0% (±0.00%)	5.0% (±0.00%)	5.0% (±0.00%)	5.0% (±0.00%)	5.0% (±0.00%)	5.0% (±0.00%)	5.0% (±0.00%)	5.0% (±0.00%)	5.0% (±0.01%)	5.1% (±0.03%)
	0.04	5.0% (±0.00%)	5.0% (±0.00%)	5.0% (±0.00%)	5.0% (±0.00%)	5.0% (±0.00%)	5.0% (±0.00%)	5.0% (±0.00%)	5.0% (±0.00%)	5.0% (±0.00%)	5.0% (±0.00%)	5.0% (±0.01%)	5.3% (±0.08%)	5.6% (±0.12%)	6.5% (±0.20%)	7.1% (±0.25%)
	0.05	5.0% (±0.00%)	5.0% (±0.00%)	5.0% (±0.00%)	5.0% (±0.00%)	5.0% (±0.00%)	5.0% (±0.00%)	5.0% (±0.00%)	5.0% (±0.00%)	5.0% (±0.01%)	5.0% (±0.01%)	5.1% (±0.05%)	6.0% (±0.16%)	6.7% (±0.22%)	7.8% (±0.31%)	8.4% (±0.34%)
	0.06	5.6% (±0.04%)	5.7% (±0.05%)	5.8% (±0.05%)	6.0% (±0.04%)	6.1% (±0.05%)	6.2% (±0.05%)	6.2% (±0.05%)	6.4% (±0.05%)	6.5% (±0.05%)	6.6% (±0.05%)	6.8% (±0.09%)	7.6% (±0.18%)	7.9% (±0.26%)	9.3% (±0.38%)	10.0% (±0.44%)
	0.07	7.9% (±0.05%)	8.0% (±0.05%)	8.2% (±0.05%)	8.3% (±0.06%)	8.5% (±0.05%)	8.6% (±0.06%)	8.7% (±0.05%)	8.8% (±0.05%)	8.9% (±0.05%)	8.9% (±0.06%)	9.1% (±0.09%)	9.6% (±0.20%)	10.3% (±0.29%)	10.8% (±0.41%)	11.8% (±0.49%)
	0.08	10.2% (±0.06%)	10.4% (±0.06%)	10.5% (±0.06%)	10.7% (±0.06%)	10.8% (±0.06%)	11.0% (±0.06%)	11.0% (±0.06%)	11.1% (±0.05%)	11.2% (±0.06%)	11.3% (±0.06%)	11.4% (±0.09%)	12.1% (±0.21%)	12.4% (±0.30%)	12.7% (±0.41%)	13.6% (±0.51%)
	0.09	12.6% (±0.06%)	12.8% (±0.06%)	12.8% (±0.06%)	12.9% (±0.06%)	13.0% (±0.06%)	13.1% (±0.06%)	13.3% (±0.06%)	13.4% (±0.06%)	13.4% (±0.06%)	13.5% (±0.06%)	13.6% (±0.08%)	13.9% (±0.18%)	14.4% (±0.28%)	15.1% (±0.43%)	15.4% (±0.51%)
	0.1	14.8% (±0.06%)	14.9% (±0.06%)	14.9% (±0.06%)	15.0% (±0.06%)	15.2% (±0.06%)	15.3% (±0.07%)	15.3% (±0.06%)	15.4% (±0.06%)	15.5% (±0.06%)	15.5% (±0.06%)	15.6% (±0.08%)	16.0% (±0.19%)	16.2% (±0.29%)	16.7% (±0.42%)	16.9% (±0.50%)
	0.15	23.3% (±0.06%)	23.4% (±0.06%)	23.4% (±0.06%)	23.3% (±0.06%)	23.4% (±0.06%)	23.4% (±0.06%)	23.4% (±0.06%)	23.4% (±0.06%)	23.4% (±0.07%)	23.4% (±0.06%)	23.5% (±0.08%)	23.7% (±0.17%)	23.9% (±0.24%)	24.4% (±0.37%)	24.5% (±0.45%)
	0.2	29.0% (±0.06%)	28.9% (±0.06%)	28.8% (±0.06%)	28.8% (±0.06%)	28.7% (±0.06%)	28.7% (±0.06%)	28.6% (±0.06%)	28.6% (±0.06%)	28.5% (±0.06%)	28.5% (±0.06%)	28.6% (±0.07%)	28.9% (±0.15%)	29.1% (±0.22%)	29.2% (±0.35%)	29.3% (±0.41%)
	0.25	32.9% (±0.06%)	32.8% (±0.06%)	32.6% (±0.06%)	32.5% (±0.06%)	32.4% (±0.07%)	32.3% (±0.06%)	32.3% (±0.06%)	32.2% (±0.06%)	32.2% (±0.06%)	32.2% (±0.06%)	32.2% (±0.07%)	32.5% (±0.14%)	32.5% (±0.19%)	33.0% (±0.29%)	32.9% (±0.36%)
	0.33	37.0% (±0.06%)	36.9% (±0.06%)	36.7% (±0.06%)	36.5% (±0.06%)	36.3% (±0.06%)	36.3% (±0.06%)	36.2% (±0.06%)	36.1% (±0.06%)	36.1% (±0.05%)	36.1% (±0.06%)	36.0% (±0.07%)	36.1% (±0.11%)	36.4% (±0.16%)	36.7% (±0.25%)	36.6% (±0.32%)
	0.5	41.7% (±0.06%)	41.6% (±0.06%)	41.4% (±0.06%)	41.2% (±0.06%)	41.0% (±0.06%)	40.9% (±0.06%)	40.8% (±0.06%)	40.6% (±0.06%)	40.6% (±0.06%)	40.6% (±0.06%)	40.6% (±0.06%)	40.8% (±0.10%)	40.9% (±0.14%)	41.2% (±0.21%)	41.3% (±0.25%)

**Table 6:** Average infected peak prevalence, varied by the infectiousness of the disease ($\beta_{se}$) and presence of hubs (network skewness) in this simulation, $\beta_{ei}$ = 0.5 (exposure to infection rate). $\beta_{ir}=0.1$, $\beta_{rs}\;=\;0$. For each parameter combination, 300 simulations were run.

		Skewness Parameter α														
		−4	−2	−1	0	1	1.5	2	2.5	3	3.2	3.5	3.8	4	4.5	5
Disease Infectiousness $\beta_{se}$	0.01	5.0% (±0.00%)	5.0% (±0.00%)	5.0% (±0.00%)	5.0% (±0.00%)	5.0% (±0.00%)	5.0% (±0.00%)	5.0% (±0.00%)	5.0% (±0.00%)	5.0% (±0.00%)	5.0% (±0.00%)	5.0% (±0.00%)	5.0% (±0.00%)	5.0% (±0.00%)	5.0% (±0.00%)	5.0% (±0.00%)
	0.02	5.0% (±0.00%)	5.0% (±0.00%)	5.0% (±0.00%)	5.0% (±0.00%)	5.0% (±0.00%)	5.0% (±0.00%)	5.0% (±0.00%)	5.0% (±0.00%)	5.0% (±0.00%)	5.0% (±0.00%)	5.0% (±0.00%)	5.0% (±0.00%)	5.0% (±0.00%)	5.0% (±0.03%)	5.0% (±0.06%)
	0.04	5.0% (±0.00%)	5.0% (±0.00%)	5.0% (±0.00%)	5.0% (±0.00%)	5.0% (±0.00%)	5.0% (±0.00%)	5.0% (±0.00%)	5.0% (±0.00%)	5.0% (±0.00%)	5.0% (±0.00%)	5.1% (±0.04%)	5.8% (±0.13%)	6.5% (±0.18%)	7.7% (±0.26%)	8.4% (±0.30%)
	0.05	5.1% (±0.02%)	5.1% (±0.02%)	5.2% (±0.02%)	5.2% (±0.03%)	5.3% (±0.03%)	5.3% (±0.04%)	5.4% (±0.04%)	5.4% (±0.04%)	5.5% (±0.05%)	5.6% (±0.04%)	5.9% (±0.09%)	7.1% (±0.21%)	7.8% (±0.27%)	9.4% (±0.38%)	10.3% (±0.38%)
	0.06	7.0% (±0.06%)	7.2% (±0.06%)	7.3% (±0.06%)	7.4% (±0.06%)	7.6% (±0.06%)	7.7% (±0.06%)	7.8% (±0.07%)	8.0% (±0.06%)	8.1% (±0.06%)	8.2% (±0.06%)	8.4% (±0.10%)	9.4% (±0.23%)	10.2% (±0.32%)	11.2% (±0.43%)	12.2% (±0.51%)
	0.07	9.9% (±0.06%)	10.1% (±0.07%)	10.2% (±0.07%)	10.3% (±0.06%)	10.7% (±0.07%)	10.8% (±0.07%)	10.9% (±0.06%)	11.0% (±0.07%)	11.2% (±0.06%)	11.2% (±0.07%)	11.4% (±0.09%)	12.0% (±0.21%)	12.9% (±0.32%)	13.6% (±0.48%)	14.2% (±0.58%)
	0.08	12.9% (±0.07%)	13.1% (±0.07%)	13.3% (±0.07%)	13.5% (±0.07%)	13.6% (±0.07%)	13.8% (±0.07%)	13.9% (±0.07%)	14.0% (±0.07%)	14.1% (±0.07%)	14.1% (±0.07%)	14.4% (±0.10%)	15.0% (±0.24%)	15.6% (±0.30%)	15.5% (±0.48%)	16.9% (±0.58%)
	0.09	15.9% (±0.07%)	16.1% (±0.08%)	16.2% (±0.08%)	16.4% (±0.08%)	16.6% (±0.07%)	16.7% (±0.07%)	16.8% (±0.08%)	16.8% (±0.07%)	17.0% (±0.07%)	17.1% (±0.07%)	17.2% (±0.10%)	17.7% (±0.23%)	18.0% (±0.33%)	19.2% (±0.52%)	19.0% (±0.57%)
	0.1	18.7% (±0.07%)	18.8% (±0.08%)	19.0% (±0.07%)	19.1% (±0.07%)	19.2% (±0.07%)	19.3% (±0.07%)	19.4% (±0.07%)	19.6% (±0.07%)	19.6% (±0.07%)	19.6% (±0.08%)	19.8% (±0.10%)	20.3% (±0.23%)	20.5% (±0.31%)	21.1% (±0.49%)	21.4% (±0.59%)
	0.15	29.9% (±0.07%)	29.9% (±0.07%)	29.8% (±0.07%)	29.9% (±0.07%)	29.8% (±0.07%)	30.0% (±0.07%)	29.9% (±0.07%)	29.9% (±0.07%)	29.9% (±0.07%)	29.9% (±0.07%)	30.0% (±0.10%)	30.4% (±0.20%)	30.4% (±0.28%)	30.5% (±0.42%)	31.2% (±0.52%)
	0.2	37.3% (±0.07%)	37.2% (±0.08%)	37.2% (±0.07%)	37.0% (±0.07%)	37.0% (±0.07%)	36.9% (±0.07%)	36.9% (±0.07%)	36.9% (±0.07%)	36.8% (±0.07%)	36.8% (±0.07%)	36.8% (±0.09%)	37.0% (±0.17%)	37.4% (±0.24%)	37.6% (±0.38%)	37.8% (±0.45%)
	0.25	42.5% (±0.06%)	42.4% (±0.07%)	42.3% (±0.06%)	42.1% (±0.07%)	41.9% (±0.07%)	41.9% (±0.07%)	41.8% (±0.07%)	41.6% (±0.07%)	41.6% (±0.07%)	41.6% (±0.07%)	41.7% (±0.08%)	42.0% (±0.16%)	42.0% (±0.21%)	42.4% (±0.33%)	42.6% (±0.40%)
	0.33	48.1% (±0.07%)	48.0% (±0.07%)	47.8% (±0.07%)	47.6% (±0.06%)	47.4% (±0.07%)	47.2% (±0.07%)	47.2% (±0.06%)	47.1% (±0.07%)	46.9% (±0.06%)	47.0% (±0.07%)	46.9% (±0.07%)	47.1% (±0.12%)	47.4% (±0.18%)	47.2% (±0.28%)	47.8% (±0.35%)
	0.5	54.8% (±0.06%)	54.6% (±0.06%)	54.4% (±0.06%)	54.2% (±0.06%)	53.9% (±0.06%)	53.7% (±0.06%)	53.6% (±0.06%)	53.4% (±0.06%)	53.4% (±0.06%)	53.3% (±0.07%)	53.4% (±0.06%)	53.5% (±0.10%)	53.9% (±0.15%)	53.8% (±0.22%)	53.9% (±0.26%)

## Discussion

With the aid of increasingly sophisticated simulation studies, social epidemiologists have begun to understand the importance that social networks play as a part of the social-structural underpinnings of numerous health outcomes [5–14].

As it pertains to the subject of this paper, there is a rapidly growing body of evidence showing that social networks shape the incidence of diseases within communities, and their spread across communities through complex dynamic transmission networks [1–4]. With the recent emergence and combination of numerous disease epidemics, scholars have begun to pay more attention to the role that larger social network structure plays in shaping disease outbreaks and spread. We build on recent work in this vein to explore how social network structure and topography might affect the spread of disease throughout the community.

## Conclusions

To begin to understand the pathways of infiltration and expanding dominion of infectious diseases throughout vast populations, social epidemiologists have focused increasingly on the topographical and structural features of the networks through which they are transmitted. It has become apparent, through network studies, that epidemic spread is a function of a complex interplay between real-world network structure and the virulence and character of a given pathogen. The present study finds that networks that have more skewed degree distributions lead to greater risk of epidemic spread overall, as has been documented in numerous other papers [49,50,99]. We expand on this work by showing that this effect depends on disease transmissibility. Our simulations show that the network-structural effect decreases as disease transmissibility increases.

What can explain this? There are several interpretations that are consistent with these findings. First, we confirm empirical and simulation evidence [79,100] that epidemics spread via hubs, but we present evidence suggesting that this effect depends on the disease’s infectiousness or transmissibility. It is possible that hubs themselves are disproportionately affected by more infectious diseases: Hubs, or “superspreaders,” as they have been called, have more exposure to people who are infected early on, and therefore are more likely to isolate early on. One recent study [101] therefore cautioned that so-called superspreaders should also be viewed as “super-receivers.” High transmissibility can effectively take such hubs out of the network, thus leaving the network disproportionately unconnected at an earlier stage in an epidemic.

An equally plausible interpretation is that any social-structural feature –social network size and topography, social resources, or other factors – simply becomes less important as diseases become more virulent. In such scenarios, networks or other social factors do not matter as much; infection and spread happens due to the sheer transmissibility of the disease. A highly transmissible disease is a disease that defies social structure and runs rampant over it.

This should not detract us from exploring further the role that social networks, contact structure and context, and heterogeneity play in shaping the epidemic spread of disease. As mentioned earlier, there is much research on how having a large number of social connections affects the spread of disease and the overall risk of epidemics. In this case, it appears that this empirical account will be more relevant when one considers less transmissible diseases or less contagious variants of diseases like COVID-19.

These findings have potential implications for the epidemic spread of diseases in certain social contexts. From a social networks perspective, these results confirm that a highly heterogeneously connected population is at greater risk for epidemic spread, especially with a relatively slowly spreading virus. Social distancing, vaccination, quarantining, and other mitigation measures can reduce the risk of epidemic spread – but these measures are particularly useful at this stage where there are numerous hubs, stars, or centers where diseases can be easily transmitted to numerous people at the same time. It is at this point that the need for public policy measures is most urgent.

### Limitations and directions

There are several limitations to this study that call for future study. For one, although maintaining a constant overall contact structure in our simulations is crucial (as this significantly influences the spread of disease), this approach may overlook the relationship between degree distribution skewness and overall contact levels. In real-world networks, total contact rate can correlate with degree distribution skewness due to factors like population characteristics and occupational distributions, so it is important to note that our findings do not take into consideration cases where both total contact and degree distribution skewness increase simultaneously.

Additionally, our results are specific to the method used to generate the simulated network(s). Discrepancies may arise when compared to other network generation methods with similar overall contact and degree distributions. Future research should evaluate our findings using different network generation methods. We did not test how different model formulations affect the impact of social network structure on spread. On one hand, due to computational constraints, among all parameters in the model we used, while varying substantively in network degree skewness and transmission rate ($\beta_{se}$) as they are core to our hypothesis, we were only able to vary two representative values for $\beta_{ei}$, three values for $\beta_{ir}$ and one value for $\beta_{ir}$ rather than fully exploring the parameter space. While this design allowed us to focus on the core mechanisms of interest, a more comprehensive sweep of parameter values would likely yield additional insights and should be pursued in future work. On the other hand, evidence suggests that the epidemic spread of disease can only partially capture models of spread by estimating $R_{\text{0}}$, using SIR or SEIR models alone, as it also requires taking into account the existence of asymptomatic, pre-symptomatic, mild, and severe categories of infection [102]. Future research should consider these different scenarios as potential conditions on the role that social network structure plays in the epidemic spread of disease. In addition, the complexity of a network’s structure and the presence of overlapping social foci or contexts make it difficult to derive a formal analytical model, leading us to rely on a series of simulations. Future work could build on this foundation by leveraging the theoretical properties of exponential distributions to develop analytical results that complement our simulation-based findings.

Our simulation parameters, while informed by documented empirical data, lack primary data on network degree distribution and variant transmissibility. As with closed-form mathematical models, questions of generalizability remain and will require careful interpretation and validation with more fine-grained contact-tracing data. Future research should track both social network structure and disease spread within a community over time and the role that network change (e.g., due to personal- and community-level network turnover) plays in shaping individuals’ susceptibility to disease and communities’ abilities to quickly organize social support, prevention, and treatment efforts [103]. However, assuming that the data upon which we build our models are reliable, we believe that our models offer valuable preliminary insights into the interplay between social network degree distribution, disease transmissibility, and epidemic spread. Expanding on this work should aide the scientific community in informing public health responses in the advent of current and future disease outbreaks, and more generally provide us with a better sense of how infectious disease transmission is rooted in social behavior and structure.

## Supporting information

S1 FigImpact of the presence of hubs/skewness in casual contact networks on peak prevalence of infection in communities by disease infectiousness (*ß*_se_) Estimates are varied by the infectiousness of the disease ($\beta_{se}$), network skewness and exposure to infectious rate ($\beta_{ei}$). In this simulation, $\beta_{ir}=\text{0}.\text{1}$, $\beta_{rs}=\text{0}$.For each parameter combination, 300 simulations were run, and 95% confidence bands are drawn.(TIFF)
