# Supplementary figures and images for: Amplification of the power of network hubs and degree skewness over infectious disease spread during lulls
Source: PLoS One. 2025 Nov 13;20(11):e0322687. doi: 10.1371/journal.pone.0322687 (PMC12614627; doi:10.1371/journal.pone.0322687)

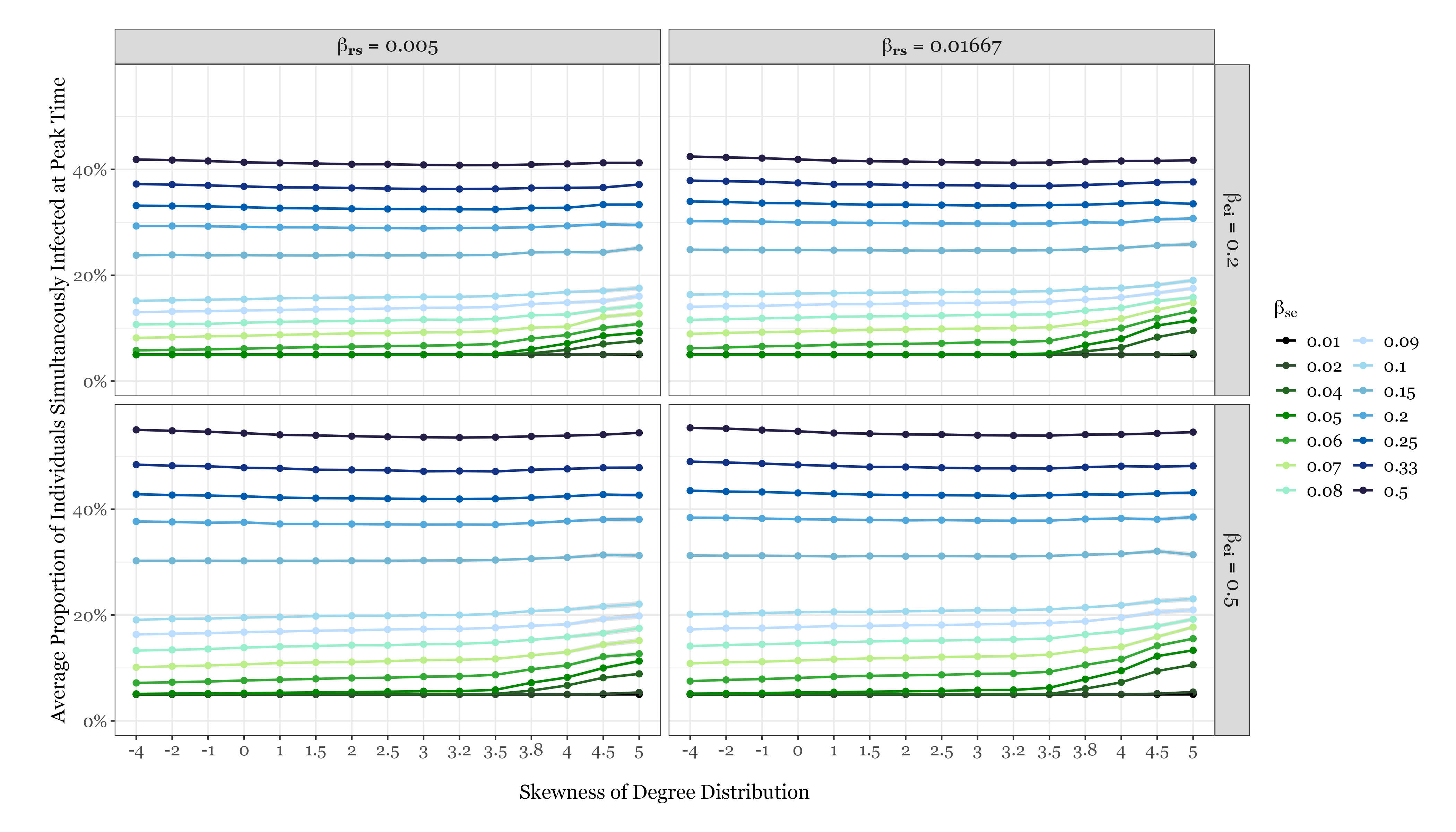

Supplement: S1 Fig — For each parameter combination, 300 simulations were run, and 95% confidence bands are drawn. (TIFF) [file pone.0322687.s001.tiff]
